# Supplementary material for: Multi‐dataset identification of innovative feature genes and molecular mechanisms in keratoconus
Source: J Cell Mol Med. 2024 Sep 19;28(18):e70079. doi: 10.1111/jcmm.70079 (PMC11412914; doi:10.1111/jcmm.70079)
Supplement: Supplementary file 2 — Material S2. [file JCMM-28-e70079-s002.zip › 7.GSEA analysis/1.ARL11/KEGG_OTHER_GLYCAN_DEGRADATION.html]

Details for gene set KEGG\_OTHER\_GLYCAN\_DEGRADATION[GSEA]

|  || Dataset | ARL11.group.cls#h\_versus\_l |
| Phenotype | group.cls#h\_versus\_l |
| Upregulated in class | l |
| GeneSet | KEGG\_OTHER\_GLYCAN\_DEGRADATION |
| Enrichment Score (ES) | -0.44020823 |
| Normalized Enrichment Score (NES) | -1.280345 |
| Nominal p-value | 0.2209073 |
| FDR q-value | 1.0 |
| FWER p-Value | 0.963 |
Table: GSEA Results Summary

  

Fig 1: Enrichment plot: KEGG\_OTHER\_GLYCAN\_DEGRADATION      
 Profile of the Running ES Score & Positions of GeneSet Members on the Rank Ordered List

  

| SYMBOL | TITLE | RANK IN GENE LIST | RANK METRIC SCORE | RUNNING ES | CORE ENRICHMENT || 1 | ENGASE | na | 9420 | 0.068 | -0.2023 | No |
| 2 | NEU4 | na | 9612 | 0.066 | -0.1416 | No |
| 3 | FUCA1 | na | 13725 | 0.021 | -0.2388 | No |
| 4 | AGA | na | 14596 | 0.014 | -0.2495 | No |
| 5 | HEXA | na | 16191 | 0.002 | -0.2930 | No |
| 6 | MAN2C1 | na | 17885 | -0.009 | -0.3322 | No |
| 7 | GLB1 | na | 18269 | -0.012 | -0.3310 | No |
| 8 | NEU1 | na | 19424 | -0.020 | -0.3442 | Yes |
| 9 | FUCA2 | na | 19891 | -0.023 | -0.3343 | Yes |
| 10 | MAN2B2 | na | 21448 | -0.035 | -0.3440 | Yes |
| 11 | NEU3 | na | 21563 | -0.036 | -0.3113 | Yes |
| 12 | HEXB | na | 22982 | -0.050 | -0.3019 | Yes |
| 13 | MAN2B1 | na | 23791 | -0.060 | -0.2649 | Yes |
| 14 | MANBA | na | 27686 | -0.123 | -0.2528 | Yes |
| 15 | NEU2 | na | 34193 | -0.451 | 0.0154 | Yes |
Table: GSEA details [plain text format]

  

Fig 2: KEGG\_OTHER\_GLYCAN\_DEGRADATION      
 Blue-Pink O' Gram in the Space of the Analyzed GeneSet

  

Fig 3: KEGG\_OTHER\_GLYCAN\_DEGRADATION: Random ES distribution      
 Gene set null distribution of ES for **KEGG\_OTHER\_GLYCAN\_DEGRADATION**
